# Foreign Correspondence

**Published:** 1854-12

**Authors:** E. Williams


### Art. VI.—FOREIGN CORRESPONDENCE.

BY E. WILLIAMS, M. D.

Vienna, Oct. 29, 1854.

Prof. Gross:—

Dear Sir, In a previous letter I gave you an account of some cases of cyst of the ovary treated in France by injections of tincture of iodine. The innocence, *in well selected cases*, as well as the utility of this treatment is fully established by the experience of those surgeons who have had recourse to it. When the proper precautions are taken to prevent the entrance of the injected fluid into the peritoneal cavity, there is very little danger of any serious consequences. If adhesion between the tumor and the walls of the abdomen exists, then, of course, there is no possibility of this accident. Should doubt exist, however, the application of caustic potash or Vienna paste to the point selected for the puncture will, in all probability, secure this adhesion.

True it is that in many cases of even very large cysts of the ovary there is not the slightest inflammatory adhesion between the peritoneal surface of the tumor and that of the abdomen or surrounding organs. At the post-mortem sections of Rokitansky I have seen several such tumors, of great size, which were entirely free from all attachments except the pedicle which unites them to the broad ligament of the uterus. As it is sometimes impossible for the most experienced surgeon to say positively whether such connections exist or not, too much caution cannot be used, and the previous application of caustic to the abdomen in all doubtful cases is advisable.

Admitting, then, that we have to do with a case of simple dropsy of the ovary, with but one, or at most few, cysts whose walls are thin and capable, after the fluid is extracted, of contracting upon each other—that the necessary adhesions exist, then certainly a recourse to repeated injections promises a radical cure without great risk of serious accidents. I am well aware that a more unfavorable state of things often exists; that the cyst is multilocular, the walls thick and sometimes containing incrustations or ossific deposits which render them incapable of contraction; or the septa may contain masses of encephaloid, or other species of cancer, as I saw in a post-mortem examination a few weeks ago. Such cases are incorrigible to every thing but *extirpation*, and between this alternative and certain death there is little ground for choice.

In the present state of the science in regard to ovariotomy, it seems to me that, in a case which is at all favorable, no one is justifiable in resorting to so shocking an expedient till he has first tried repeated injections. In case of failure no harm is done, and the unwelcome resource of a vivisection is still at the disposal of the surgeon, who then takes up his bistoury with the full conviction that nothing else can save his patient. So far as I know the method of injections has not yet been tried either in England or America.

During the months of March and April, which I passed in London, I was cognizant of two patients who fell victins to the time-sanctioned, but none the less revolting, procedure of extirpation, by which every tyro in the profession now seeks to make himself the subject of a newspaper puff. A blind and servile adherence to the rules and forms prescribed by the fore-fathers in the profession is as prejudicial to the interests of humanity as to the progress of medical science. Give me the daring, energetic spirit of Dupuytren, who made war upon nearly all the received doctrines of his day, and enriched the science with so much that was new and original, in preference to that of Boyer, who rejected, without trial, everything that was not sanctioned by the authority of his predecessors. If innovation in surgery promises relief without subjecting the patient to a species of living vivisection little preferable to death, why in the name of humanity let us give it a trial!

The first case I saw was in the University College hospital. It was a woman thirty five years of age, the mother of several children. The tumor of near three years standing, had increased to such an extent that, three months before her entrance into the hospital, a puncture was necessary. A large quantity of fluid was drawn off, and the tumor, for the time, *entirely disappeared, showing it to be a simple cyst with thin walls.* At the time of her appearance at the hospital the abdomen had again acquired its previous very large size. The surgeon, Mr. Erichsen, resolved, after a careful examination, to attempt entirpation. At that time the uterus was drawn up so that its fundus was distinctly felt through the walls of the abdomen in the left hypogastric region, forming a small firm tumor fixed to a larger fluctuating one which lay more towards the right side. The os uteri was so high as to be reached with difficulty by the finger in the vagina. On introducing a sound into the bladder it passed high into the abdomen before its point was arrested by the fundus of the organ, which was drawn up and flattened out on the anterior surface of the cyst. The dragging up of the uterus and bladder out of the pelvic into the abdominal cavity, seemed to me only explicable by the supposition that they were strongly adherent to the tumor, and formed, to my mind, a great objection to any attempt at extirpation. The surgeon, however, did not so consider it, and stated to his class, before commencing the operation, that he hoped there were no adhesions so extensive as to prevent the complete removal of the cyst, but that it was impossible to say before opening the abdomen, whether it was free from attachments or not.

An incision three inches long was made in the median line, commencing a little below the umbilicus and extending towards the symphisis pubis, but the *adhesions* of the tumor with the uterus, bladder, and in the right iliac and hypogastric regions were so extensive and firm as to render the completion of the operation impossible! The cyst was incised, its contents evacuated, and the opening attached, by strong sutures, to the lips of the wound in the abdominal parietes, which latter was closed by sutures and adhesive strips. About forty-eight hours afterwards, if I mistake not the time, the patient died of peritonitis, an event not at all astonishing.

Several days before the operation, Mr. Erichsen, who, I must say, is an excellent operator and a very clever man, had the kindness to show me the patient and spoke of his intention to perform ovariotomy. I suggested the propriety of injections of tincture of iodine, and related to him what I had seen of the results of that treatment. He replied that it had not yet been *resorted to in England*, and he feared it might give rise to *grave peritonitis.* To the latter objection it was only to be answered that the experience of French surgeons had proved it to be infinitely less dangerous to life than any attempt at extirpation. As to the former, *its not yet having been tried in England*, that was sufficient to determine the choice of the more classic and imposing method of laying open the abdomen!

The second case occurred at Guys hospital a few days afterwards. The account of it I have from a medical gentleman who was present at the operation. All the surgeons of the distinguished "corps of Guys hospital," were firmly settled in the conviction that the tumor was free from adhesions, and consequently a favorable one for extirpation. An incision was made, but, to the astonishment of all the worthy successors of Sir. Astley Cooper, it was found so *extensively and firmly adherent* that the operation was abandoned. A portion of the cyst only was excised and the wounds in the abdomen united as in the previous case. After four or five days the patient sank from general peritonitis.

It is very true that there are many cases on record of remarkable recoveries after extirpation, but the multitude of tentatives which have been followed by results similar to the above, is so great as to make the conscientious surgeon shrink from the responsibility of so formidable an undertaking till all other means are exhausted, and nothing remains but to choose between ovariotomy and certain death. Every case of success is blazoned forth to the world through all the journals in Christendom, while the great majority of failures are unfortunately withheld from public scrutiny. Hence the statistics on this subject, unfavorable as they are, would be far more so if the whole truth were fairly and candidly stated.

I have neither time, space nor the inclination to give you a succinct account of the *great* hospitals and surgeons of the *great* metropolis of London—a city which has been the theatre of action of the most remarkeble names that adorn the history of our noble science. When I, for the first time, traversed the courts, museums, wards and operating rooms of those majestic hospitals, I felt that I was treading upon consecrated ground. In consequence of its antiquity and historical associations, all that I saw possessed for me charms, and a degree of importance, doubtless somewhat above its intrinsic worth. And when ushered into the presence of the renowned, living representatives of HUNTER, COOPER, KEY, LISTON, and a host of others, whose fame knows no limits but those of civilization, and no end but the end of time, you can easily imagine that a modest man like myself experienced a feeling of extraordinary humiliation! But with time and observation this embarrassing spell happily vanished, and I was reminded of the fact that if familiarity does not always breed contempt, it at least modifies and restrains within due bounds the exaggerated ideas which we, as *too servile* American physicians, often entertain of our far-famed brethren across the Atlantic!

Freed from the enchantment which *distance lends to the view*, I have, with the eye of an impartial, and I hope, not entirely incompetent observer, viewed the medical giants of Paris, London, and Vienna, (as well as many others of less celebrity), without being able to discover in what particular they so wonderfully surpass many members of our *young and energetic profession in the young but giant America.* American energy and activity are proverbial all over Europe, and if we only had the hospital and other advantages which she as yet enjoys over us, the New World would soon number as many celebrated names and perhaps more than the Old.

Mr. FERGUSSON enjoys by far the greatest reputation of any surgeon in London. By birth he is a Scotchman and it is to his indomitable energy of character that he owes his extraordinary success in the profession. So far as pecuniary matters were concerned, he commenced his studies *poor*, but his natural genius united with untiring application has placed him at the head of the surgical galaxy of England. Since his removal from Edinburgh to London however, in 1840, many other things have conspired to his extremely rapid elevation, particularly the death of so many of his renowned competitors. As the stars of Sir. ASTLEY COOPER, LISTON, KEY and others sank below the horizon of eternity, that of FERGUSSON rose with successively increased brilliancy and is now perhaps in the zenith of its glory. He is only forty-six years of age and possesses a practice and reputation of which medical history furnishes us few examples. It is said that he now realizes, at least, £10,000 annually from his practice. He is a large, robust man at least six feet high, and in his countenance is depicted a singular combination of benevolence and good humor with unflinching determination. He is, without instituting any invidious comparisons, the most beautiful operator that I have ever seen. Besides many trivial operations, I saw him perform lithotomy, amputation of the thigh immediately below the trochanter major, in consequence of an encephaloid tumor of the fermur, and amputation of the forearm. I was present at the concluding lecture of his course in Kings College, and witnessed him repeat the amputations on the *dead subject.* In these he appeared to manifest great "*sang froid*," amounting even to *indifference* for his subject and his *flaps*, the latter of which did not always come off without *holes;* and I was surprised to see the amount of pure *physical force* used, and of which he possessed no ordinary amount. Mr. FERGUSSON is decidedly a *bad lecturer*, as every one who has ever heard him, I am sure, will testify. In his position and gesticulations awkward, he hesitates and hems and grunts to such an extent that it is absolutely painful to hear him. In consequence of his affability and politeness towards his medical cotemporaries, he enjoys a large circle of warm personal friends and fewer enemies than most men who have attained to such an enviable position in the profession. In conclusion I venture the remark that Mr. FERGUSSON's operative skill has contributed more to his great fame than his original researches or profound knowledge of the science of surgery.

Mr. LAWRENCE who has written perhaps the best existing treatise on hernia and also a voluminous work on the eye, is still one of the surgeons to St. Bartholomew's hospital. He is now quite old and operates but little. I saw him operate upon one case of strangulated inguinal hernia and was really surprised at the precision and steadiness of his hand. He is still affable, gay, and lively as if in the prime of life.

Mr. STANLEY, of the same hospital, author of the admirable treatise on the bones, is also somewhat advanced in years. In his levity and talkativeness as well as personal appearance, he approaches more nearly the French than the English character. On learning that I was from the *States* (as they say in London) he manifested toward me much politeness and attention. One day, after having spent an *hour and a half*, without the slightest manifestation of impatience, in operation for an ununited fracture of the femur, in which he cut down upon the ends of the fragments, bored holes through them, hammered in ivory pegs and united them by a silver wire, he turned to me and asked how we treated these "nasty, vexatious ununited fractures in the States?" Among other things I mentioned to him the new method, first proposed and executed by Prof. Brainard of Chicago, of perforating the ends of the fragments in different directions by a species of stab-awl introduced through the soft parts, and then applying an apparatus so as to maintain entire immobility of the limb. Prof. Brainard maintains, from numerous experiments on animals, that foreign bodies in contact with bone prevent, instead of promoting, the formation of callus. "Well, well," said he, "that is something new to us in London. I must confess, you Yankees are a sharp set of fellows." I beg your pardon, Mr. Stanley, but that is a rather equivocal compliment. "How so sir?" First, Every American does not feel himself flattered by the appellation of *Yankee*, especially the backwoodsmen, of which I am one; and, second, if one with us succeeds well in the manufacture and traffic of *wooden nutmegs* we call him a *sharp fellow*. "O, I beg ten thousand pardons sir, I mean *ingenious*, *thorough-going fellows*." I relate this little anecdote because it illustrates the hilarity and good humor of the man.

Mr. Paget, lecturer on physiology in St. Bartholomew's hospital, perhaps thirty-eight or forty years old, is emphatically one of those lean and hungry-looking men who think instead of sleep of nights. His reputation, as surgeon, is rapidly rising. His researches in regard to the structure, &c., of the different varieties of cancer are the result of much reflection and observation. So far as mere cutting is concerned, he is surpassed by many younger men in London who have not the tithe of his experience and sound judgment. Mr. Paget is in every respect a polite and clever gentleman and reminds me much of your former colleague, Prof. Cobb.

In University College Hospital the principal surgeons are Mr.

Erichsen and Mr. Quain; one of the editors of Quain and Sharpeys anatomy. The former a young and promising surgeon, operates a great deal, and has written recently a treatise on surgical pathology. As to Mr. Quain, he is, to use a vulgar but very applicable expression, a "dry stick." It is said that he has not smiled in the last half century. I saw him but once and that for me was sufficient. An infant was brought into the amphitheatre suspected of imperforate rectum. A number of students and several strangers were there curious to know the result of his examination. He examined carefully the child, without seeming to be cognizant of the presence of a living soul, and then sent it away. The deathly silence of the occasion was not disturbed by a single word from the sage professor. After this came his visit in the wards where he ligatured two hemorrhoidal tumors and reduced a paraphymosis, without uttering a half dozen words. This melancholy scene I had no curiosity to witness a second time.

With Mr. Guthrie, I had the pleasure of a short interview. He is affable but dignified in his conversation, and his presence inspires that respect and admiration that age and wisdom never fail to command. Like Mr. Lawrence, however, he is fast upon the wane. The climax of his reputation has been long since attained. His son, one of the surgeons to the Westminster Ophthalmic hospital, would certainly have acquired greater fame as a horse-jockey than as ophthalmologist, had he turned his attention in that way.

In Guy's hospital are several surgeous of considerable reputation but no one of them stands out so preëminently above the others as to merit any special remarks. In this famous hospital there is at this time certainly no Sir. Astley Cooper, although in its surgeon's staff may be numbered some promising men.

Mr. Simon, of St. George's Hospital, is very rapidly rising in the esteem and confidence of the profession. If not already, he will in a few years certainly, be the first pathologist and one of the best surgeons of England. In personal appearance he is by no means prepossessing, and his voice, because decidedly nasal, is unpleasant. Although, perhaps, not more than thirty eight or forty years of age, protracted study and anxiety have impressed upon his naturally feeble frame marks of a much more advanced age. I witnessed several of his operations, some amputations, extirpation of tumors and ligature of the femoral artery for the relief of popliteal aneurism. Only once did I see him break over the limits of his usual calmness and self-possession, qualities so essential to a good operator. During an effort to remove a sequestrum of the condyle of the femur, the patient having lost much blood and being under the influence of chloroform, swooned, and for some seconds appeared to be really dead. He threw violently down his hammer and chisel, sprang to the table, seized a pitcher of water, dashed it in the patient's face and yelled at the top of his voice "*ammonia, ammonia, d—n it have you no ammonia?*" This indiscribably ludicrous scene, especially the *nasal twang, sui generis*, with which he cried for the ammonia and his agonizing gesticulations, excited to such an extent my risibility that I was compelled to make an almost equally desperate struggle to suppress a violent explosion of laughter, solemn as was the occasion.

At the London hospital, in consequence of its proximity to the docks, are treated an immense number of accidents, especially fractures and dislocations. I was much astonished at the wonderful rapidity with which fractures heal in this institution. In no other hospital in Europe have I seen such prompt and favorable results. This is due in part, doubtless, to the extreme cleanliness observed in all the wards, but more especially to the fact that from almost the very commencement the patients are feasted upon rich soups, roast beef and porter.

This system of diet contrasts as strikingly with the *soupe maigre, eat-nothing*, system adopted in the Parisian hospitals, as do the results of the two methods. The frequency of gangrene, purulent infection, diarrhœa, &c., following accidents and operations, is incontestably much greater in Paris than in London.

Mr. Adams, of the London hospital, is a prudent and cautions surgeon and operates very finely. On the 18th of March last, I saw him perform disarticulation of the femur in three-quarters of a minute, and with the loss of scarcely more than an ounce of blood. The patient died seven days after the operation, of diarrhœa and general prostration. Mr. Adams stated that just eighteen years previonsly the thigh was successfully disarticulated in the same hospital. No case of recovery, I believe, has ever been published in France.

M. Maisonneuve, of Paris, became in some way possessed, last winter, of the wonderful notion that those cases of sudden death from what he calls *gangrène fondroyatne*, after accidents and operations, are caused by the rapid development of gas in the veins and its entrance into the circulation. A few days after he had conceived this idea, in traversing his wards in hospital Cochin, he came to a patient whose leg had been amputated a few days previonsly, put his hand upon the stump, stroked it from above downward, and behold there issued from the flabby wound a *bubble of gas.* He called instantly for a knife and in less than a minute from the time this unfortunate bubble was seen the patient's thigh was removed at the hip joint. The patient being *moribund* at the time of the operation died the same day. An unlucky *globule* had probably already been wafted above poupart's ligament, out of the reach of his knife.

I did not visit the numerous other general hospitals in London sufficiently often to be able to give you an account of them. Besides these there are many *special institutions;* for the treatment of diseases of the eye, in particular. Of the latter, the one which most attracted my attention, and which I visited every day during two months, is the Royal London Ophthalmic Hospital. The building itself is not imposing. Besides the waiting hall, the general reception room, a committee and an operating chamber, are only a few small rooms containing in all about twenty-five beds for the reception only of patients subjected to an operation, or to whom some accident has happened to the eye requiring the recumbent position. All others are prescribed for as *out-door* patients, of whom something more than one hundred are daily waited upon and supplied with medicine.

Belonging to the hospital are five surgeons and one physician. Each day, from 9 to 11 o'clock, two of the surgeons are in attendance, and prescribe for all *really indigent* patients who apply. Nothing can exceed the order with which this admirable institution is conducted, and no where during my voyage have I seen such a vast amount of material for the study of ophthalmology.

This hospital, as well as many others in London, is supported by voluntary contributions, a fact which speaks loudly for the benevolence of the English people.

Mr. Bowman, (of Todd and Bowman's Anatomy and Physiology), is one of the surgeons. He is a rather small man, perhaps forty years of age, modest and unassuming in his manners, disposed only to talk when he has something very important to say, of an exceedingly pleasant countenance, but one which indicates at the same time profundity of thought and reflection. He has devoted much of his well-directed attention to the anatomy and physiology of the eye, upon which subject he is every where received as one of the very best authorities. He operates upon the eye with a degree of delicateness and dexterity which I have seldom had occasion to witness. Accuracy of diagnosis and precision of hand, the two great essentials for an ophthalmic surgeon are qualities for which he is much celebrated. Of some ideas and operations peculiar to him I shall have occasion to speak at some future day; at present I have no time.

Mr. James Dixon, another of the distinguished surgeons of this hospital, is somewhat younger in the profession than Mr. Bowman, but very rapidly rising in reputation. Among the few acquaintances which I made in London, it was Mr. Dixon with whom I was the most pleased. Polite without ostentation, affable and talkative without loquacity, in his bearing dignified but easy and attractive, he is much admired and esteemed by all who have had the pleasure of his acquaintance. As to stature he is rather a large man and possessed in every way of a fine personal appearance. It is especially in the private circle that his qualities are displayed to the best advantage, and that he infuses into all around him a feeling of ease, comfort and hilarity. For some time past Mr. Dixon has gradually disembarrassed himself of general surgical practice and devoted his undivided attention to ophthalmology, in which he has acquired a large experience. He has at his disposal a very rich material for the study of diseases of the eye and no one certainly, knows better how to render it useful. He has written a book entitled "Guide to the study of Eye diseases," which will appear in a few months, and which, judging from his experience, patience and accuracy of observation, will be a valuable acquisition to the science. Among many other operations I saw him extirpate the lachrymal gland in consequence of an extensive burn involving the skin of the whole side of the face, followed by a cicatrix that caused incurable ektropion. There was incessant "larmoiment" which greatly annoyed the patient, and which to a great extent was relieved by the operation. I think he has performed, and with favorable results, this rare operation three times, which is so generally condemned by surgeons as unjustifiable and useless.

Mr. Critchel and Mr. Wordsworth are the two youngest surgeons of this hospital. They are good operators, ardent lovers of the science, patient and accurate observers, and in every way worthy of the high station they have attained not only as surgeons to the Ophthalmic, but also to the London Hospital.

Mr. McMurdo, the oldest surgeon of this institution, is more remarkable for his vanity, ostentation, pendantry and hostility to tobacco than for sound judgment or operative skill.

In conclusion I must say that the hospitals of London in external appearance and especially in internal accommodations are superior to all others in Europe. As the stranger struggles and dodges and elbows his way along the million thronged thoroughfares of this vast metropolis and scans the majestic edifices dedicated to the relief of human suffering, that tower up so imposingly on all sides, he needs no other argument to satisfy him that the Englishman's cold and formal exterior must conceal a warm and noble heart that throbs with active, *pocket-reaching*, sympathy for the miseries of his fellow beings.

On my way from England to Vienna I stopped a few days in Brussels, particularly to visit the Ophthalmic Institute which enjoys quite a reputation in Europe. I saw there many exceedingly interesting cases which I have no time now to describe; among others several who had been subjected to inoculation with the actual pus of gonorrhœa, for the relief of pannus, with most beautiful results.

M. Warlomont, (editor of the *Annales d'Oculistique*,) informed me that he had experimented with direct inoculation upon some thirty patients, and that in nearly all the pannus, as well as the granular lids, which nearly always either cause or accompany it, was speedily cured without serious accidents. He does not at all combat the inflammation which follows the inoculation. In a small monograph he has published (or soon will) an account of his tentatives.

In Paris I witnessed the treatment, by inoculation, of one patient who had suffered for years with trachoma and pannus. On the second day as soon as acute inflammation arose the lids were cauterized with nitrate of silver. The patient was speedily and readily cured.

Arrived in Vienna the 18th of May, I remained there near five months in attendance at the great general hospital with its vast materials and celebrated professors. This immense building was founded by the Emperor Joseph II, and erected at the expense of the government. Till two years ago the so called *General Hospital* consisted of three departments. 1st. The Allgemeines Kraukenhaus, (common hospital). 2d. The Lying-in Hospital with the Foundling house. 3d. The Insane Hospital, (a very antique looking, perfectly circular, building with very small windows and resembling more a vast tower with port holes than any thing I can imagine). A new Insane Hospital has recently been erected in a remote situation, and is the most beautiful and commodious building of the kind I have ever seen. The foundling house is now also removed to another locality, so that at present the general hospital consists of the common and the lying-in hospitals. The entire building of the general hospital, covering an area of more than ten acres of ground, consists of ten quadrangles enclosing numerous large courts ornamented with trees, promenades and benches for the use of convalescents, and is capable of accommodating, in case of necessity, 3500 patients, though there are seldom more than 1500 or 2000 in the wards at once. In the whole hospital are treated yearly from 20,000 to 30,000 patients. The medical faculty of the University of Vienna has control, through stipulated annual payments, of certain divisions, but not of the whole hospital. They have three medical, two surgical, one eye, and one midwifery clinic; a department for diseases of women, one for syphilis and one for diseases of the skin. In the other part there are two surgical divisions, one for diseases of the chest, one for nervous diseases and a peculiar lying-in establishment. There are three classes of patients in the hospital. The first pays one guilder and thirty kreutzers daily, for which he has a small room, a nurse, good eating and can *choose* his physician. The second pays forty-eight kreutzers daily. Of these twelve are lodged in one large room and supplied with two or three waiters. The third class pays thirty kreutzers and are distributed off in large numbers together and supplied with a limited number of nurses. The second and third classes have not the right to choose their physician but must be content with the one appointed for the division. A patient who presents a certificate from the police or from his employer, that he is unable to pay, is received and the expenses charged upon the parish or upon the master. Persons born in Vienna or having resided in the city ten years are only charged half of the above rates.

The Director of the Hospital is appointed by decree of the Emperor and has a salary of 3,000 guilders annually. The Professors, the Primarie, the Secundarie and Assistants, by the Minister of Instruction. The salaries of the Professors vary according to the extent of their reputation, &c., &c. The lowest is 1600 guilders, the highest, I believe, 6000 annually. In addition the Professor also receives from each student, for Lemester of six months, as many guilders as he gives hours weekly, in lecturing. Each Assistant receives 460 guilders yearly, with the privilege of giving private instruction, for which he can charge what he will. No Assistant can hold his place more than four years. There are besides, several *extraordinary* Professors who give clinical lectures but receive no salary except what they charge each student who attends.

The Lying-in Department is a part of the general hospital and is divided into three parts. 1st. The clinic for the instruction of physicians. 2nd. That for midwives, (females). 3rd. The "*Secret*," or paying division. The clinic, destined for the practical instruction of physicians, consists of five wards in the first, and four in the second story, containing one hundred and twenty beds. Women far advanced in pregnancy, or actually in labor, are received here during four days in the week; the other three days being devoted to the department for midwives. The visit of the professor, with clinical instruction, occurs each day from 8 till

11 o'clock, A. M., and all students, who pay 4 guldens quarterly, have the privilege of attending and touching, under the professors supervision. At the evening visit, generally held by the assistant, a limited number of students are also admitted and exercised in touching. Each day two students, under the name of *journalists*, are appointed, whose duty it is to remain day and night in the lying-in chamber and examine all women received. Ten regularly graduated and installed midwives aid in the natural deliveries.

The clinic for the education of female midwives, is quite separated from the other, but conducted in the same way. Young ladies who design becoming midwives are here instructed in all that pertains to that branch, daily exercised in touching, and at the end of four months, after a thorough examination, receive diplomas. In these two clinics are matured yearly, from 150 to 200 *male* and from 260 to 300 *female* midwives. During the year 1853, 4221 women were delivered in the clinic for physicians, and 3480 in that for midwives, in all 7701, being by far the largest number of *accouchments* that occur in any one such establishment in the world. All the children at the age of a few days, are sent to the foundling hospital, put out to hired nurses, and at length, when old enough, either taught a useful trade or made soldiers. At least three-fourths of them, however, die within the first few years.

In the *secret* or paying division women who are able to pay a certain sum daily, may be received and delivered in the most clandestine manner. Their names are given *under seal* only to be opened in case of death, and those of the first class can, if they choose, wear masks or veils. Their presence is kept the most profound secret, and no one under any condition is allowed to enter this department, except the appointed physician and the nurses. The fact also of their having been there is not allowed to be proved in a court of justice. Into the policy, in a moral point of view, of this establishment I shall not now enter. There are many good arguments pro and con.

A few words in regard to some of the most celebrated professors in the Vienna school and I will afflict you no more. Prof. ROKITANSKY, born in Bohemia of poor parents, is about 48 years old. He is a large, robust man who enjoys in a high degree, good living and good society. He has a large head with a high, broad forehead, prominent cheek-bones, wide mouth and eyes that indicate more of benevolence and good humor than vivacity. The portrait which I will bring you will give you a better idea of his appearance than any description from my pen. Prof. Rokitansky made his studies in Prague and in Vienna. Appointed assistant in 1827, of pathological anatomy, extraordinary professor in 1836, he was elevated to the grade of Professor in 1845, so that the high position he has attained, and his world-wide fame as pathological anatomist have been reached only through many years of patient and laborious study. All legal sections in Vienna and precincts are made by him, without exception, and amount yearly to about 500. For this he receives a fixed annual salary of 600 guilders. His salary as Professor is 3000 guilders and lodging. Besides this he receives 8 guilders each Lemester, from every student who receives instruction from him in his peculiar branch, amounting to from 2200 to 2400 guilders, also every candidate whom he examined pays 10 guilders, making yearly from 1000 to 1500. He is nominally Prosector of the hospital, but his assistants make all the autopsies, which reach annually from 1500 to 2000. In the afternoon, three times a week, he gives instruction to students in the dead house, on pathological anatomy and the method of conducting post-mortem examinations. Prof. Rokitansky has written, besides his great work on Pathological Anatomy, several very valuable monographies—one on struma, another on the structure of cancer, one on colloid and villous cancer, and a treatise on the most important diseases of the arteries, with plates. The latter has been recently published, and is a most invaluable book for every one who feels an interest in surgery or pathological anatomy. The plates are most admirable. In the museum of pathological anatomy, I have examined carefully all the preparations of which he speaks, and can therefore judge of the accuracy of his plates and descriptions. A new edition, entirely revised, of his Pathological Anatomy, with plates, is now being published. The first volume will appear in December of this year. This new work will be much better than the first, but from what I can learn I do not believe it will be fully up to the present state of the science. His only competitor in pathological anatomy in all

Germany, worthy of the name, is Prof. VIRCHOW, of Wirthsburg. Many consider him now superior to ROKITANSKY. Between them no very kind feelings exist. Each has his party of admirers and calumniators as does every other renowned professor in Germany. In all respects, ROKITANSKY is ths dryest, dullest, most uninteresting lecturer that I have ever heard. Nothing but the most intense interest in the subject and absolutely heroic courage can enable any one to resist the narcotizing influence of his tone and appearance, for an hour. Candor compels me to acknowledge that all my energies failed.

Prof. SKODA is also a Bohemian by birth, and descended from poor parents—is now forty-two years old, unmarried, very rich, drinks enormous quantities of *beer* and suffers much from *gout.* He made his studies in Vienna, was first appointed Secundarius in the department for skin diseases, then Primarius, and finally, in 1847, clinical Professor, and became the most renowned physician in Austria. Since the publication of his work on auscultation and percussion, he has abandoned hard study. A new edition of his book is just published. His practice is confined almost entirely to consultation cases for which his fee varies from 5 to 200 guilders. Outside of the profession of medicine, SKODA is not a learned man, and it is particularly in diseases of the chest that his reputation has been attained. In private society his presence overwhelms every soul with the deepest melancholy. Apart from his speciality he is, in all respects, as dry and uninteresting a being as it is possible to imagine. He lectures almost eternally upon the organs contained in the cavity of the chest, and always in that dull, sing-song, monotonous voice which is peculiar to himself, and which no one, who has still a particle of fire and energy in his soul, can endure without the greatest pain. There he stands by the bed-side—a pair of spectacles across his nose, the glasses of which approach the size of a common saucer, his eyes forever fixed upon a point of the floor slightly in front of his great toe, a pleximeter in one hand and the little hammer, armed with indian-rubber, with which he thumps the patients, in the other—for half or three-quarters of an hour, talking in this dry, never-changing tone, and then prescribes *Aqua Laurocerasi*, and walks slowly and quietly to another bed to repeat the same solemn ceremony. In accuracy of diagnosis Prof. Skoda is perhaps unequalled, but he has not the slightest confidence in the efficacy of medicines, hence the *peculiarity* of his prescription.

Prof. Oppolzer the other lecturer on clinical medicine is, on the contrary, extremely affable, easy and attracting in his manners, and one of the most interesting lecturers to whom I have had the pleasure to listen. He was born in Prague and is now forty-six years of age. He was formerly assistant of the late celebrated clinical professor, Krompholz, in Prague, and through whom he first became known. Through his private courses he soon acquired a great name as clinical lecturer, was called to Leipsic as Professor, and in 1849 to Vienna where he receives an annual salary from the State of 5,000 guilders. He is an extremely popular professor and has the largest consultation practice in Vienna. He is a man of a most extraordinary memory, very amiable in every respect, but of no great scientific value to the profession in general. He has written little or nothing. In his manner of dressing he, as well as Skoda, is extremely careless—absolutely *slovenly.*

Hyrtl, professor of anatomy in the University of Vienna, of perhaps the same age, is a man of great learning, much natural genius, and is certainly the most interesting lecturer in the faculty He is the son of a violin-maker in Vienna, and was remarkable, during his studentship, for being the greatest rake in the whole country. He lectures on general, descriptive, surgical and comparative anatomy, and makes the most beautiful anatomical preparations I ever saw. Prof. Hyrtl was formerly very rich, but in 1848 almost his entire property with his museum of human and comparative anatomy, was burned by the soldiers. In his private character he is sometimes very sociable and polite, at others exceedingly peevish and morose and secludes himself from society.

On a future occasion I will perhaps write you something about Prague and Berlin. Next winter I hope to see you. Adieu.

